# Atypical presentation of sector retinitis pigmentosa

**DOI:** 10.5935/0004-2749.2025-0031

**Published:** 2025-09-10

**Authors:** Mariana Calheira Gontijo, Mariana Gouveia Bastos Meirelles, Ricardo Luz Leitão Guerra

**Affiliations:** 1 Leitão Guerra Oftalmologia, Salvador, Bahia, Brazil

A 58-year-old woman presented with bilateral, asymmetric sector retinitis pigmentosa (RP) (Figure A, B). Fundus autofluorescence imaging revealed hyperautofluorescent rings surrounding hypoautofluorescent regions corresponding to bone spicule pigmentation and retinal degeneration (Figure C, D). The left eye also exhibited nuclear cataract–induced media opacities. Despite the asymmetry, macular integrity was relatively well-preserved in both eyes. Asymmetric and sector RP is a rare variant of the disease characterized by localized retinal involvement, typically affecting specific quadrants and displaying less symmetry compared with the classic diffuse form of RP^(1^,^2)^.

**Figure:**
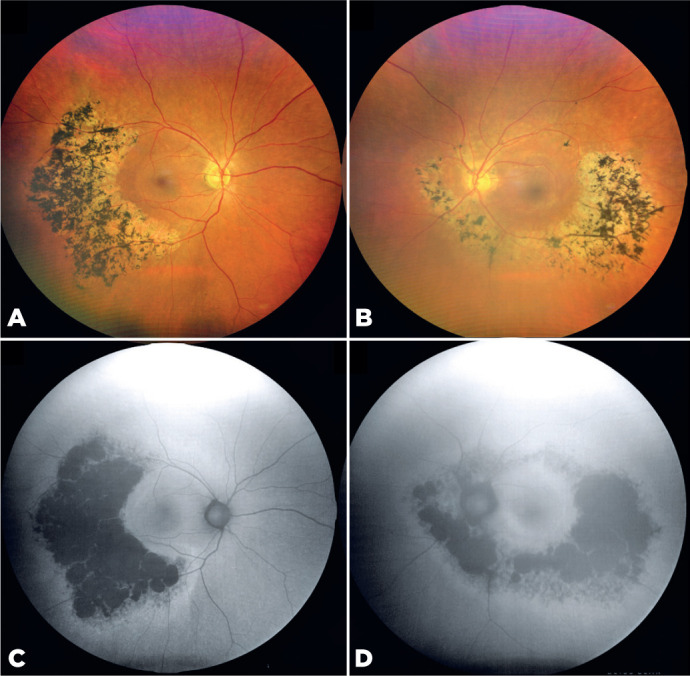

## Data Availability

All data generated or analyzed during this study are included in this published article.
